# Effect of tumor necrosis factor-α on the expression of the ammonia transporter Rhcg in the brain in mice with acute liver failure

**DOI:** 10.1186/s12974-018-1264-8

**Published:** 2018-08-22

**Authors:** Wen Wang, Hui Lu, Xu Lu, Donglei Wang, Zhaohan Wang, Wenying Dai, Jinyong Wang, Pei Liu

**Affiliations:** 1 Department of Infectious Diseases, The First Affiliated Hospital of China Medical University, 155 Nanjing North Street, Shenyang, 110001 Liaoning Province People’s Republic of China; 2 Gastroenterology and Hepatology Department, Jiangxi Provincial People’s Hospital, Nanchang City, Jiangxi Province People’s Republic of China; 3 Department of Intervention, the Sixth People’s Hospital of Shenyang, Shenyang City, Liaoning Province People’s Republic of China; 4 The Institute of Liver Diseases of China Medical University, Shenyang, Liaoning Province China

**Keywords:** Acute liver failure, Hepatic encephalopathy, Ammonia transporters, Tumor necrosis factor-alpha, Blood brain barrier

## Abstract

**Background:**

Ammonia and tumor necrosis factor-alpha (TNF-α) play important roles in the mechanisms of hepatic encephalopathy (HE). Rhesus glycoprotein C (Rhcg) is important for ammonia transport especially in the kidney. The aim of the present study was to investigate the role of Rhcg in the brain in acute liver failure (ALF) and the effect of TNF-α on Rhcg expression.

**Methods:**

ALF mouse models were generated by treatment with d-galactosamine (D-GalN) and lipopolysaccharide (LPS), or D-GalN and TNF-α. ALF induction was blocked by pretreatment with anti-TNF-α IgG. The levels of serum TNF-α were determined by ELISA. Blood ammonia and brain ammonia concentrations were detected using an ammonia assay kit. The expression and distribution of Rhcg in the brain tissues of ALF mice were examined by western blotting, real-time PCR, immunohistochemical, and immunofluorescence analyses.

**Results:**

Serum TNF-α levels were increased in the LPS/D-GalN group. Blood and brain ammonia were increased in the LPS/D-GalN- and TNF-α/D-GalN-induced ALF groups. Rhcg mRNA and protein levels were elevated in both ALF groups, consistent with the increase in blood and brain ammonia. Rhcg was mainly expressed in vascular endothelial cells and astrocytes. Pretreatment with anti-TNF-α IgG antibody downregulated Rhcg in brain tissues in the LPS/D-GalN group, prevented the occurrence of ALF, and reduced blood and brain ammonia levels in the LPS/D-GalN group.

**Conclusion:**

TNF-α promoted the transport of ammonia from the blood to brain tissues and exacerbated the toxic effects of ammonia by upregulating Rhcg.

## Background

Acute liver failure (ALF) is the clinical manifestation of sudden and severe hepatic injury and arises from many causes. ALF patients develop hepatic encephalopathy (HE) and subsequently symptomatic cerebral edema (CE), which progresses to intracranial hypertension. Although the incidence of clinically overt CE has decreased over the past 20 years, such hypertension still accounts for 20–25% of deaths [1, 2]. Survival without transplantation is poor in patients with ALF complicated by severe encephalopathy.

Despite several decades of investigation, the mechanisms responsible for HE associated with ALF remain incompletely understood. Ammonia is universally considered the key precipitating factor that drives cerebral dysfunction in HE. In patients with ALF, the plasma concentrations of ammonia are drastically elevated and related to the development of brain edema and herniation [3, 4]. A longstanding hypothesis proposes that the development of CE is related to the cerebral uptake of ammonia from the blood and subsequent disturbance of the intracellular metabolism [4]. This raises the question of how ammonia enters the brain, and how the cerebral uptake of ammonia is affected.

The conventional assumption is that unionized ammonia (NH_3_) can pass the blood–brain barrier by diffusion, whereas translocation of the ionized species (NH_4_^+^) can be neglected. However, the so-called diffusion hypothesis is controversial [5, 6]. Evidence suggests that a variable fraction of the ammonia uptake occurs through channel- or carrier-mediated transport. Recently, human nonerythroid Rhesus (Rh) glycoprotein B (RhBG) and nonerythroid Rh glycoprotein C (RhCG), as well as their non-human mammalian homologs (Rhbg/Rhcg), which are members of the Amt/MEP/Rh family, were identified as mammalian ammonium/ammonia transporters [7]. Rhbg and Rhcg are widely expressed on the plasma membranes of various mammalian organs such as the liver, kidney, gastrointestinal tract, and lung [8–12]. Rhbg transports both NH_3_ and NH_4_^+^, whereas Rhcg only transports NH_3_ [13]. Epithelial expression of RhCG mRNA has been reported in the testis, prostate, pancreas, and brain [8], and Rhcg plays a critical role in ammonium handling and pH homeostasis both in the kidney and in the male reproductive tract [13, 14]. Whether Rhcg is located at the luminal or abluminal side of brain capillaries and its capacity for brain ammonia transport remain unclear.

In addition, the correlation between the severity of HE and ammonia concentration is not always consistent in patients with ALF or chronic liver failure [15–17]. Compared with placebo/no intervention, ammonia-lowering strategies are associated with beneficial effects on HE in patients with chronic liver diseases [18, 19], whereas these agents are much less effective in ALF [20]. These observations have led to a search for additional or alternative causes of brain edema and HE in ALF.

Evidence suggests that, in ALF, ammonia and proinflammatory cytokines act synergistically to affect brain function and cause HE and brain edema [21–25]. TNF-α plays an important role in ALF from various causes. A close association between the severity of HE and the circulating levels of TNF-α is reported in ALF patients and animal models [24–27]. At the cellular level, TNF-α increases ammonia uptake in human cerebrovascular endothelial cells [28] and causes cell swelling in ammonia-sensitized cultured astrocytes [29].

The aim of this study was to investigate the expression of the ammonia transporter Rhcg in the brain tissues of an ALF mouse model and the effect of TNF-α on brain Rhcg expression.

## Methods

### Animals and experimental protocols

Male C57BL/6 mice (6–8 weeks old, Animal Laboratory, China Medical University, Shenyang, Liaoning Province, China) were maintained on a 12:12-h light/dark cycle in a temperature-controlled, air-conditioned room, with free access to food and water. The mice were divided into eight groups. One group received intraperitoneal injections of d-galactosamine (D-GalN, 300 mg/kg body weight, Sigma, St. Louis, MO, USA) and lipopolysaccharide (LPS) (*Escherichia coli* O111:B4, 5 μg/kg body weight; Sigma) to induce ALF. A second ALF-induction group received intraperitoneal injections of D-GalN at 600 mg/kg body weight and TNF-α (10 μg/kg body weight, Origene, Rockville, MD, USA). Five control groups were intraperitoneally injected with D-GalN at 300 or 600 mg/kg body weight, LPS at 5 μg/kg body weight, TNF-α at 10 μg/kg body weight, or normal saline. One group was treated with anti-TNF-α–IgG antibody (Sigma) at 5 μg/g body weight via vena caudalis injection 30 min before LPS + D-GalN injection. Mice were euthanized 12 h after the administration of LPS/D-GalN. Mice were fasted for 12 h before drug injection. The eight groups were as follows: (1) LPS/D-GalN, (2) TNF-α/D-GalN, (3) D-GalN 300 mg/kg control, (4) LPS 5 μg/kg control, (5) TNF-α 10 μg/kg control, (6) saline control, (7) D-GalN 600 mg/kg 12 h control, and (8) anti-TNF-α–IgG antibody + LPS/D-GalN 12 h control. Mice from groups 1–5 were euthanized at 2, 6, 9, 12, and 24 h, mice from groups 6–8 were euthanized at 12 h. There were five mice at every time point in every group. Mice were sacrificed by decapitation, and serum was isolated from blood samples obtained by eyeball extirpation; brain and liver tissues were stripped for subsequent analyses. The study was approved by the Ethics Committee of China Medical University.

### Blood biochemistry and serum TNF-α assay

Serum levels of alanine transaminase (ALT) were determined using an automatic analyzer (Hitachi, Tokyo, Japan). Serum TNF-α levels were determined using an ELISA kit (eBioscience, San Diego, CA, USA) according to the manufacturer’s protocol.

### Histopathological analysis of liver tissue and brain tissue

Liver tissues were fixed in 10% phosphate-buffered formalin, and brain tissues (frontal parietal lobe) were fixed in 4% paraformaldehyde solution, embedded in paraffin, and cut into 5-μm serial sections using a rotatory microtome. Sections were stained with hematoxylin-eosin.

### Measurement of blood and brain ammonia concentrations

Blood and brain ammonia concentrations were determined using an ammonia assay kit (BioVision, Milpitas, CA, USA) according to the manufacturer’s protocol.

### Western blot analysis of Rhcg in brain tissues

To detect Rhcg proteins, brain tissue samples were extracted in RIPA lysis buffer, followed by centrifugation (12,000×*g*, 30 min). Protein concentration was determined with a BCA protein assay reagent kit (Thermo Fisher Scientific, Waltham, MA, USA). Equal amounts of proteins (100 μg) were separated by 8% SDS-PAGE and electrophoresed. Proteins were transferred onto a polyvinylidene difluoride (PVDF) membrane (Millipore, Billerica, MA, USA), which was blocked with 5% non-fat dried milk in Tris-buffered saline (TBS) containing 0.05% Tween-20 (TBST) for 1 h at room temperature. After blocking nonspecific antibody binding, the PVDF membrane was incubated with mouse anti-mouse monoclonal Rhcg antibody (1:1000, Sigma) at 4 °C overnight. After washing with a TBST three times to remove unbound antibody, the membrane was incubated with a 1:2000 dilution of goat-anti-mouse IgG conjugated to horseradish peroxidase (HRP) for 1 h at room temperature. The membrane was washed three times with TBST to remove unbound secondary antibody and developed with a luminol chemiluminescent detection kit (Thermo Fisher Scientific). Protein expression was quantified by densitometry. GAPDH was used as an internal loading control. Relative protein levels were calculated based on a densitometric count ratio of Rhcg over GAPDH.

### Rhcg RNA isolation and real-time quantitative polymerase chain reaction

Total RNA was isolated from mouse brain tissues using TRIzol reagent (Takara, Dalian, China). The concentration and purity of mRNA were determined by spectrophotometry at 260 and 280 nm, and mRNA was diluted to 500 ng/μL with DEPC water. RNA was incubated at 37 °C for 15 min, followed by 85 °C for 5 s for reverse transcription (RT) (PrimeScript RT Master Mix; Takara) to generate cDNA. The cDNA underwent 45 cycles of PCR (95 °C for 30 s, 95 °C for 5 s, and 60 °C for 30 s) [SYBR Premix Ex Taq II (Tli RNaseH Plus)] in the TP800 Thermal Cycler Dice Real Time System (Takara). The primers used were as follows: Rhcg forward, 5′-GCGCTGTAGGCTTCAACTTC-3′ and reverse, 5′-GGCTGACCTTGCCTAGAACT-3′; GAPDH forward, 5′-TCTCTGCTCCTCCCTGTTCC-3′ and reverse, 5′-GTTCACACCGACCTTCACCA-3′. The length of the PCR products for Rhcg and GAPDH were 183 bp and 91 bp respectively. Relative levels of Rhcg gene expression were calculated based on the amplification of a standard curve after a series of cDNA dilutions. GAPDH was used as a housekeeping control. Each sample was tested in triplicate.

### Immunohistochemistry and immunofluorescence analysis for brain Rhcg

Mouse brain sections (frontal parietal lobe) were fixed in polyformaldehyde, embedded in paraffin wax, sectioned, and mounted on coated slides. Immunolocalization of Rhcg was accomplished using immunoperoxidase procedures. Brain sections were dewaxed in ethanol, rehydrated, and then rinsed in phosphate-buffered saline (PBS). To observe Rhcg localization, brain sections were incubated for 3 min in citrate buffer pH 6.0 in a pressure cooker at 100 kPa. Endogenous peroxidase activity was blocked by incubating the sections in 3% H_2_O_2_ for 10 min. The sections were blocked for 10 min with 10% normal goat serum (Zhongshan Biotechnology, Beijing, China) and then incubated overnight at 4 °C with a dilution of 1:300 of primary Rhcg antibody (Sigma). The sections were washed in PBS and incubated for 10 min with polymer-linked, peroxidase-conjugated goat anti-rabbit IgG (Zhongshan Biotechnology, Beijing, China); washed with PBS; and then exposed to diaminobenzidine for 5 min. The sections were washed in distilled water and then dehydrated with xylene, mounted, and observed by light microscopy. Image analysis was perfomed using Image J and Rhcg expression was quantified as % of IHC-colored pixels of the cerebral cortex.

To investigate the localization of Rhcg in the cerebral cortex, its colocalization with glial fibrillary acidic protein (GFAP, an astrocyte marker), CD31 (a marker of vascular endothelial cells), and neuronal nuclei (NeuN; a marker of neurons) was examined by immunofluorescence detection. Paraffin-embedded coronal sections were dewaxed with ethanol and rehydrated, and then placed in repair boxes filled with EDTA antigen retrieval buffer (pH 6.0). Antigen retrieval was performed in a microwave oven. After they cooled naturally, the sections were rinsed with PBS, blocked for 30 min with bovine serum albumin (Servicebio, Wuhan, China), and incubated overnight at 4°C in a humidified chamber with mouse monoclonal anti-Rhcg (Santa Cruz Biotechnology, Santa Cruz, CA, USA; 1:100), rabbit polyclonal anti-CD31 (Abcam, Cambridge, UK; 1:200), rabbit polyclonal anti-GFAP (Proteintech, Wuhan, China; 1:1000), or rabbit polyclonal anti-NeuN (Proteintech; 1:200) antibody diluted in PBS. After washing in PBS, sections were incubated for 50 min at room temperature with a fluorophore-tagged species-specific secondary antibody (Cy3-conjugated goat anti-mouse IgG at 1:300 or AF488-labeled goat anti-rabbit IgG at 1:400; both from Servicebio). After washing with PBS, the sections were stained with 4',6-diamidino-2-phenylindole (DAPI) (Servicebio; 1:200) for 10 min at room temperature. After several washes with PBS, the sections were mounted with fluorescence mounting medium (Servicebio) and protein colocalization was examined by confocal microscopy (Eclipse C1, Nikon, Tokyo, Japan).

### Statistical analysis

The software package SPSS, version 20.0, was used for statistical analysis. All data were analyzed using analysis of variance (ANOVA) followed by a least significant difference (LSD) test. *P* values of < 0.05 were considered significant. All data are presented as the mean ± standard deviation (SD).

## Results

### Effect of TNF-α in mice with LPS/D-GalN-induced acute liver failure

**Fig. 1:**
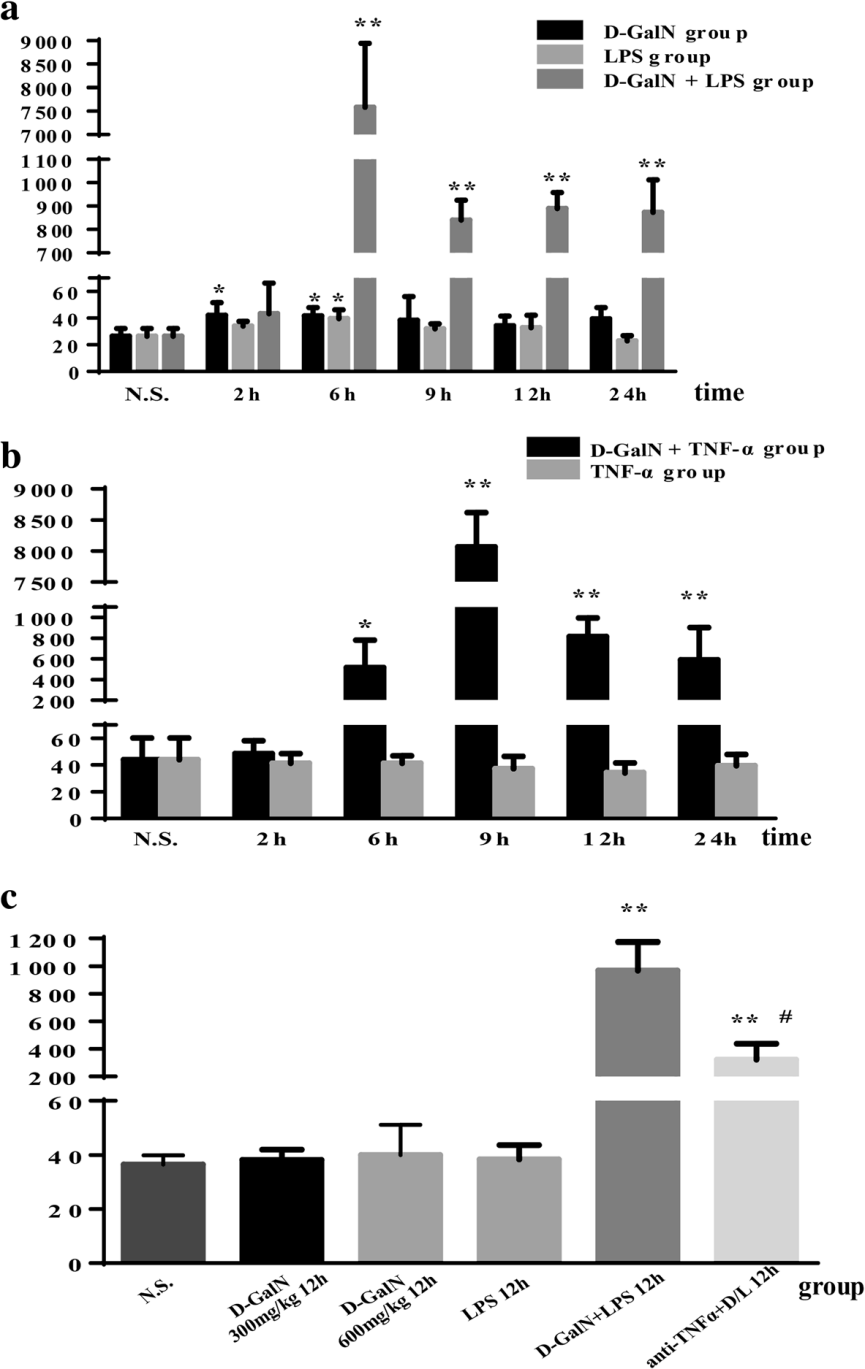
Serum levels of ALT (U/L). **a**, **b** Serum ALT levels were significantly higher in the LPS/D-GalN group (**a**) and TNF-α/D-GalN group (**b**). **c** In the anti-TNF-α IgG group, serum ALT levels decreased rapidly compared to those in LPS/D-GalN-treated mice. Data represent means ± SEM (*n* = 5 per group). *P < 0.05 vs. N.S. group, **P < 0.01 vs. N.S. group, #*P* < 0.01 vs. D-GalN+LPS 12 h group. ALT alanine transaminase, D-GalN d-galactosamine hydrochloride, LPS lipopolysaccharide, TNF-α tumor necrosis factor-alpha, N.S. normal saline

Mice gradually developed poor appetite and activity after 4 h of treatment with LPS/D-GalN, resulting in convulsions and coma, followed by death starting at 5 h. The mortality rate was 28% (18/65) at 6 h, 50.7% (33/65) at 9 h, and 60% (39/65) at 12 h. There were no additional deaths between 12 and 24 h. Serum ALT levels were significantly higher in the LPS/D-GalN group than in the saline controls beginning at 6 h post injection, reaching 7587.00 ± 1354.76 U/L (*P* < 0.01 vs. saline controls). Serum ALT levels were lower at 9, 12, and 24 h than at 6 h but significantly higher than those in the saline controls (*P* < 0.01 vs. saline controls). There were no deaths in the D-GalN control, LPS control, and saline control groups, and mice showed normal activity within the 24-h observation period. Serum ALT levels were in the normal range in D-GalN controls and LPS controls, although they were higher than those in the saline controls at several time points (Fig. 1a).

**Fig. 2:**
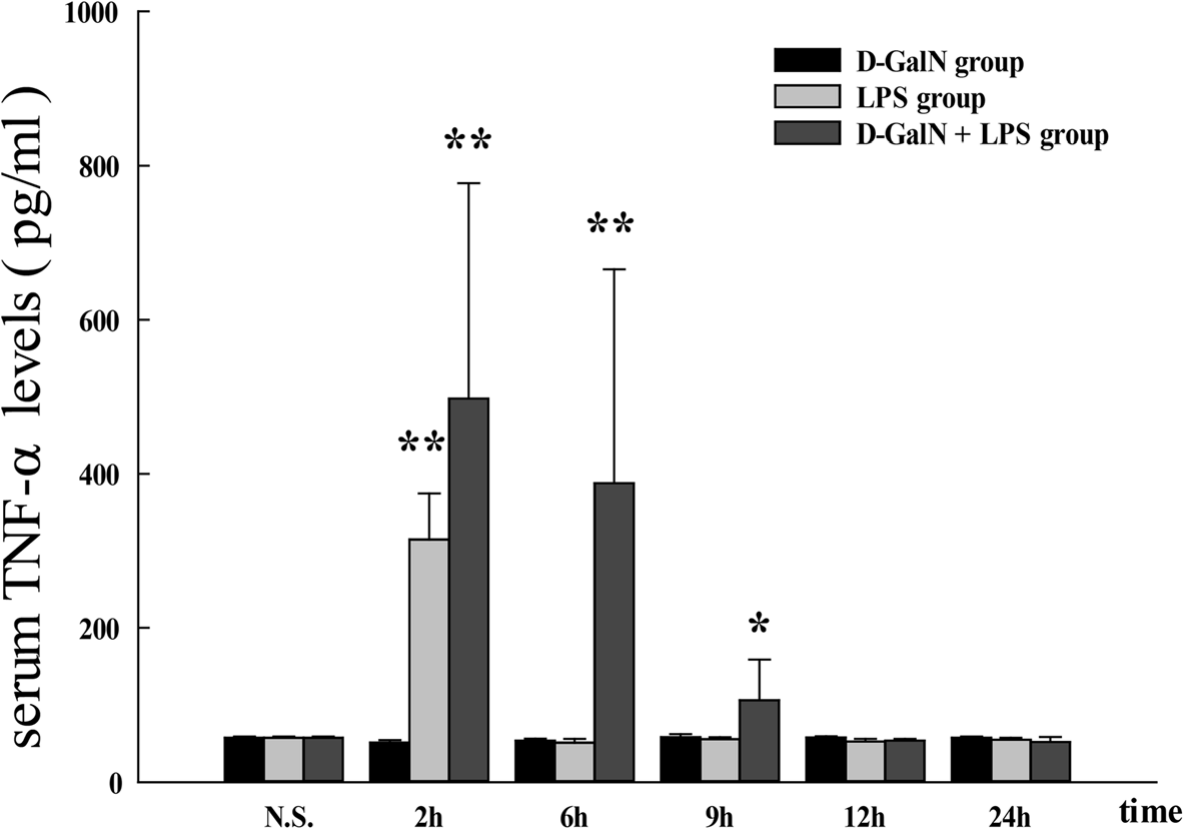
Serum TNF-α levels in LPS/D-GalN-induced ALF group and controls. Serum TNF-α levels were increased in response to LPS/D-GalN stimulation, reaching a maximum at 2 h post injection, followed by a gradual decrease at 6 and 9 h and reaching normal values at 12 and 24 h. In the LPS control group, serum TNF-α levels peaked at 2 h and declined markedly to a normal level at 6 h. There were no differences in serum TNF-α level between D-GalN and saline controls. Data represent the mean ± SEM (*n* = 5). *P < 0.05 vs. N.S. group. **P < 0.01 vs. N.S. group. ALF acute liver failure, N.S. normal saline, D-GalN d-galactosamine hydrochloride, LPS lipopolysaccharide, TNF-α tumor necrosis factor-alpha

Serum TNF-α levels in saline controls were 57.62 ± 1.74 pg/mL and significantly increased in response to LPS/D-GalN, reaching a maximum of 497.37 ± 279.94 pg/mL at 2 h post injection (*P* < 0.01 vs. saline controls), followed by a gradual decrease, with values of 388.08 ± 277.28 pg/mL at 6 h (*P* < 0.01 vs. saline controls) and 112.51 ± 46.90 pg/mL at 9 h (*P* < 0.05 vs. saline controls). TNF-α levels decreased to normal values at 12 and 24 h. In the LPS control group, serum TNF-α levels reached a peak level of 314.91 ± 59.76 pg/mL (*P* < 0.01 vs. saline group) at 2 h and declined dramatically to the normal value at 6 h. There were no differences in serum TNF-α levels between D-GalN controls and saline controls (Fig. 2).

Because TNF-α is potentially involved in the pathogenesis of ALF, we used TNF-α to replace LPS for inducing ALF in conjunction with D-GalN. Mice showed poor appetite and activity after 5 h of treatment with TNF-α/D-GalN, resulting in convulsions and death starting at 7 h. The mortality rate was 66.7% (50/75) at 9 h and 80% (60/75) at 12 h. There were no more deaths between 12 and 24 h. There were no deaths in the D-GalN 600 mg/kg 12 h controls, TNF-α controls, and saline controls within the 24-h observation period. In the TNF-α/D-GalN group, serum ALT levels increased significantly to 516.20 ± 265.01 U/L at 6 h (*P* < 0.05 vs. saline group) post injection, reaching a maximum value of 8067.20 ± 551.78 U/L (*P* < 0.01 vs. saline controls) at 9 h. Serum ALT levels were lower at 12 and 24 h than at 9 h, although they were significantly higher than those in the saline controls (*P* < 0.01 vs saline controls) (Fig. 1b).

**Fig. 3:**
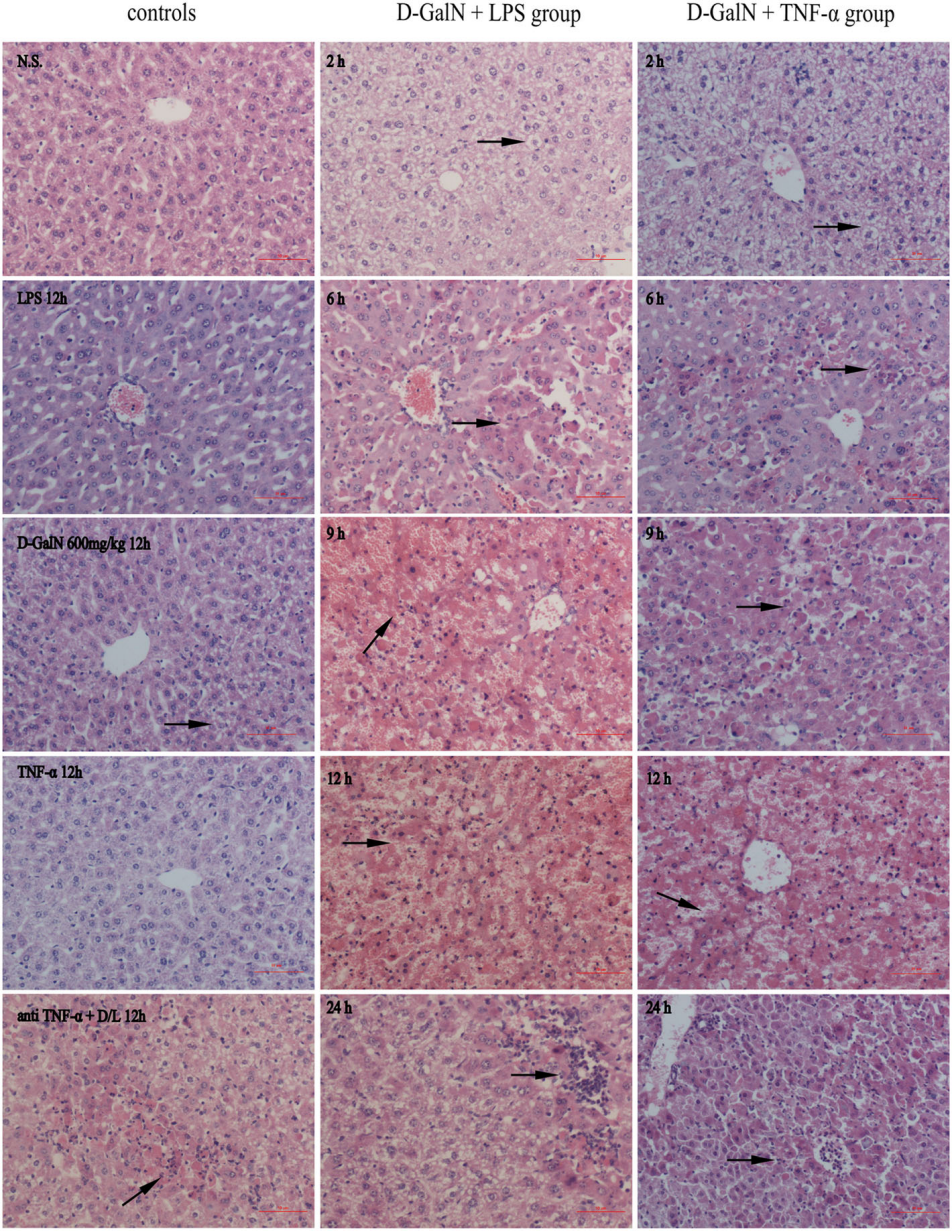
Representative images of hematoxylin and eosin-stained mouse liver tissue specimen (× 200 original magnification). Severe hepatocyte swelling, congestion, and focal necrosis (arrows) were observed 6 h post LPS/D-GalN injection. Massive or submassive necrosis of liver tissues (red areas) was observed at 9 and 12 h post LPS/D-GalN injection and at 12 h post TNF-α/D-GalN injection. Only mild-to-moderate hydropic degeneration of hepatocytes (arrows) was observed in D-GalN and LPS controls. Mice in the anti-TNF-α group showed only hydropic degeneration of hepatocytes or focal-hepatonecrosis (arrows). Scale bar 10 μm. N.S. normal saline, D-GalN d-galactosamine hydrochloride, LPS lipopolysaccharide, TNF-α tumor necrosis factor-alpha

Liver histopathology showed severe hepatocyte swelling, congestion, and focal necrosis at 6 h post LPS/D-GalN injection. In addition, massive or submassive necrosis of liver tissues was observed at 9 and 12 h post LPS/D-GalN injection (Fig. 3). There was only mild-to-moderate hydropic degeneration of hepatocytes in D-GalN controls and LPS controls (Fig. 3). Mice injected with TNF-α/D-GalN showed similar histopathological characteristics to those of the LPS/D-GalN group (Fig. 3). The most severe pathological changes occurred at 12 h post TNF-α/D-GalN injection, as shown by massive or submassive necrosis of liver tissues.

Because 60% (39/65) of mice with LPS/D-GalN-induced ALF died within 12 h and displayed serum biochemical markers and liver morphology consistent with liver failure, we chose a 12-h time point to assess the effect of anti-TNF-α IgG antibodies on liver failure. No deaths occurred among the five mice in the treated groups. Serum ALT levels decreased rapidly compared with those in LPS/D-GalN-treated mice (321.60 ± 116.01 U/L vs. 970.00 ± 205.81 U/L, *P* < 0.01, Fig. 1c). Histopathological examination showed only hydropic degeneration of hepatocytes or focal-hepatonecrosis (Fig. 3).

### Effect of TNF-α in mice with hepatic encephalopathy during acute liver failure

**Fig. 4:**
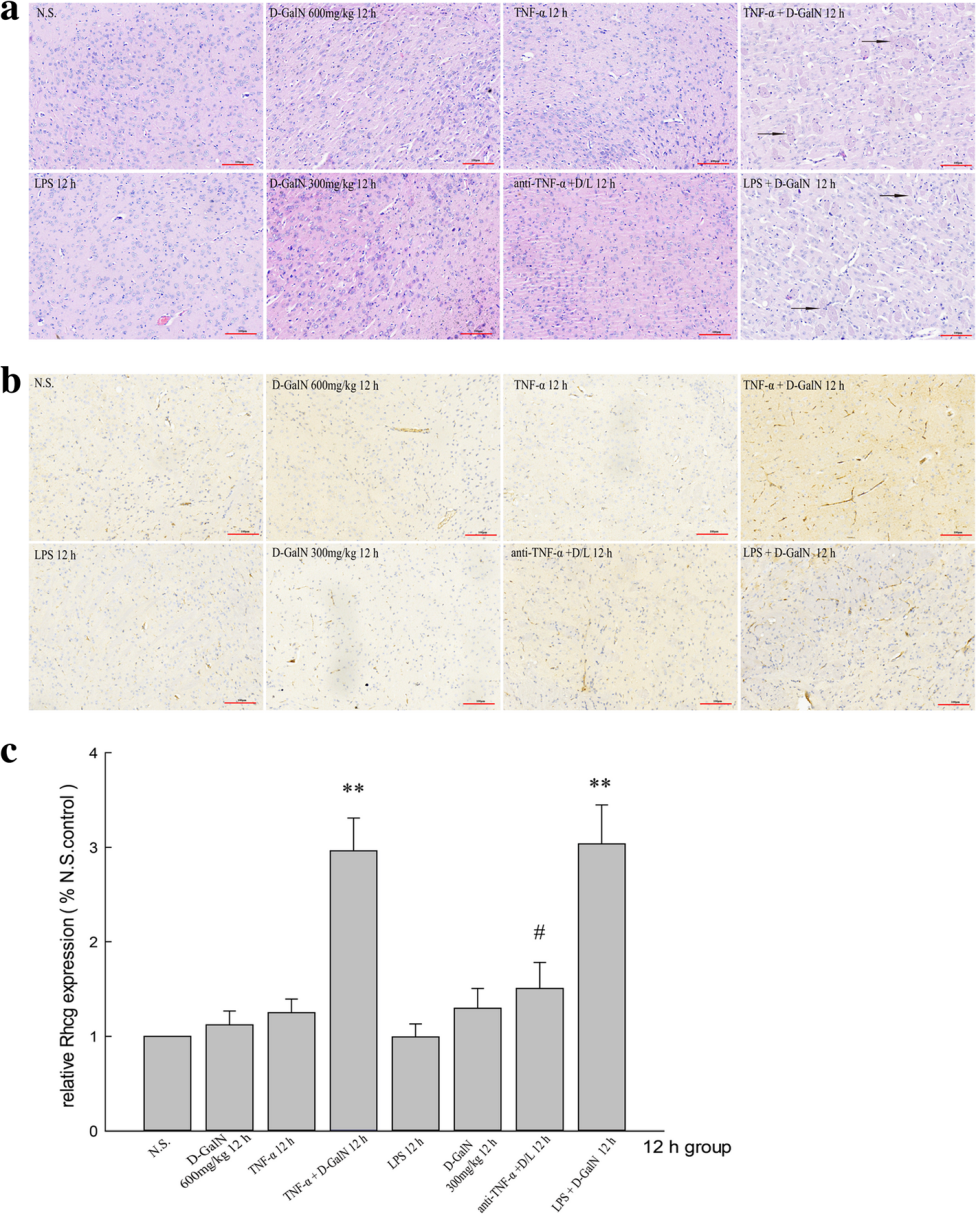
Representative images of hematoxylin and eosin-stained mouse frontoparietal brain specimens and immunohistochemical detection of Rhcg. (× 200 original magnification). **a** Hydropic degeneration and edema of cortical neurons was observed 12 h post LPS/D-GalN and TNF-α/D-GalN injection; this was accompanied by a larger cell size and diffuse and pale cytoplasm (arrows). There were no obvious changes in other controls, including the anti-TNF-α + LPS/D-GalN group. **b**, **c** Immunohistochemical analysis detected strong Rhcg expression in brain tissues in the LPS/D-GalN- and TNF-α/D-GalN-induced ALF groups. Treatment with anti-TNF-α–IgG antibodies reduced Rhcg expression in the LPS/D-GalN group. Scale bar 100 μm. Data represent means ± SEM (*n* = 5 per group). **P < 0.01 vs. N.S. group, #*P* < 0.01 vs. D-GalN+LPS 12 h group. N.S. normal saline, D-GalN d-galactosamine hydrochloride, LPS lipopolysaccharide, TNF-α tumor necrosis factor-alpha

A histopathological analysis of brain tissue (Fig. 4a) showed hydropic degeneration and edema in the cerebral cortex brain tissues at 12 h post LPS/D-GalN injection and TNF-α/D-GalN injection. Cells were enlarged and the cytoplasm diffuse and pale-colored. There were no changes in brain tissue appearance in D-GalN, LPS, and TNF-α control and the anti-TNF-α-treated group.

**Fig. 5:**
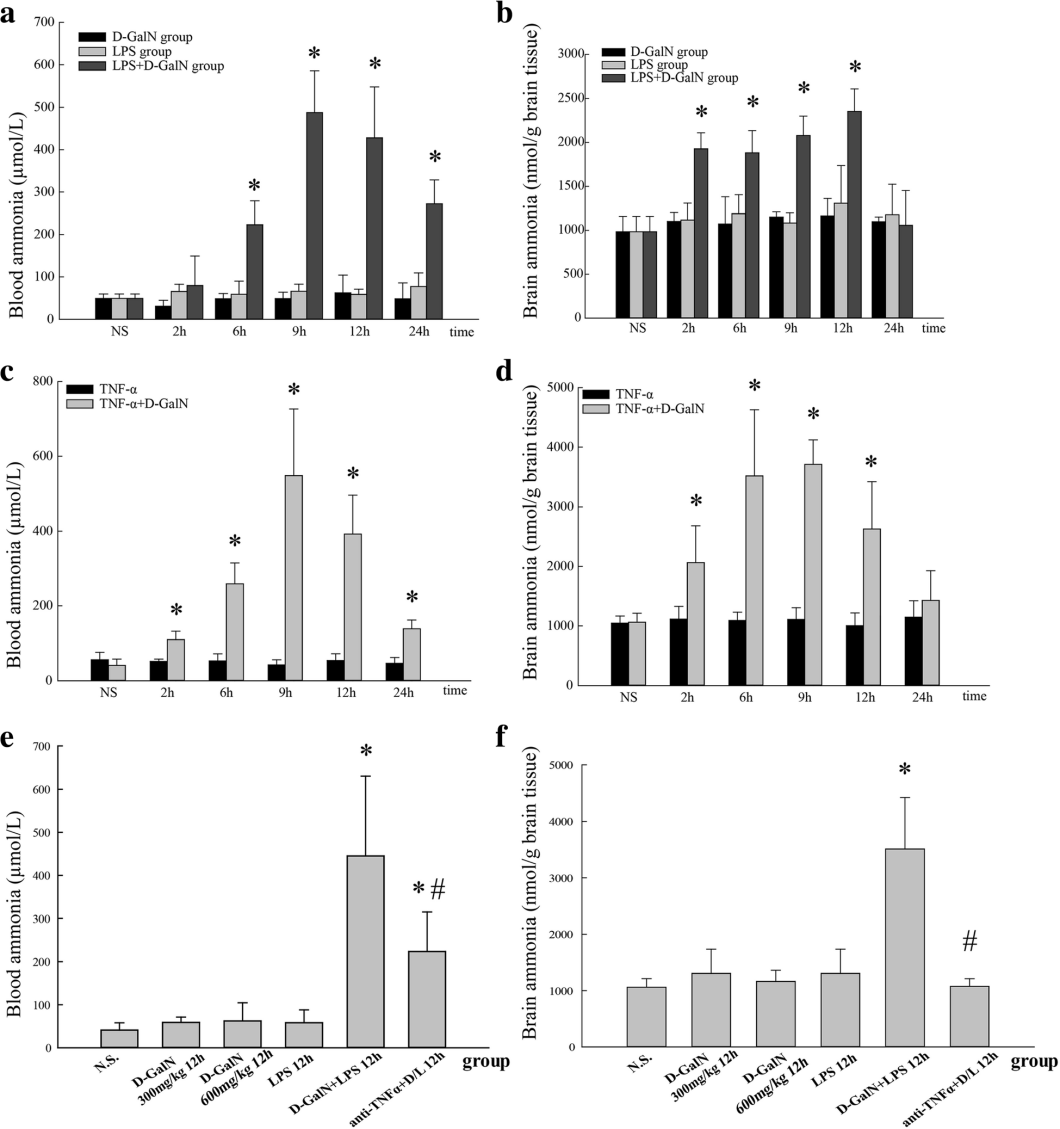
Blood and brain ammonia concentrations. **a** Blood ammonia concentrations in the LPS/D-GalN, D-GalN control, and LPS control groups. **b** Brain ammonia concentrations in the LPS/D-GalN, D-GalN control, and LPS control groups. **c** Blood ammonia concentrations in the TNF-α/D-GalN and TNF-α control groups. **d** Brain ammonia concentrations in the TNF-α/D-GalN and TNF-α control groups. **e** Blood ammonia concentrations in the anti-TNF-α–IgG + LPS/D-GalN and other control groups at 12 h. **f** Brain ammonia concentrations in the anti-TNF-α–IgG + LPS/D-GalN and other control groups at 12 h. Data represent means ± SEM (*n* = 5 per group). *P < 0.01 vs. N.S. group, #*P* < 0.01 vs. D-GalN+LPS 12 h group. N.S. normal saline, D-GalN d-galactosamine hydrochloride, LPS lipopolysaccharide, TNF-α tumor necrosis factor-alpha

Blood ammonia and brain ammonia concentrations are shown in Fig. 5. In mice injected with LPS/D-GalN, blood ammonia levels did not change at 2 h compared with those in the saline group (79.66 ± 69.80 vs. 49.09 ± 10.62 μmol/L, *P* > 0.05). Ammonia levels began to increase at 6 h (223.15 ± 56.21 vs. 49.09 ± 10.62 μmol/L, *P* < 0.01) and reached peak levels at 9 h (487.45 ± 98.13 μmol/L) and 12 h (427.95 ± 120.27 μmol/L). Blood ammonia levels at 24 h were decreased compared with those at 12 h, although they were significantly higher than those in the saline controls (272.39 ± 56.28 vs. 49.09 ± 10.62 μmol/L, *P* < 0.01) (Fig. 5a).

Brain ammonia levels began to increase at 2 h in the LPS/D-GalN group (1927.26 ± 182.76 vs. 984.11 ± 174.49 nmol/g brain tissue, *P* < 0.01), increased to 1881.89 ± 252.35 nmol/g brain tissue at 6 h and 2078.83 ± 221.53 nmol/g brain tissue at 9 h, and reached the highest level at 12 h (2352.92 ± 257.55 nmol/g brain tissue, *P* > 0.05 vs. 9 h group, *P* < 0.01 vs. other groups), followed by a decrease to normal level at 24 h (1057.24 ± 394.63 nmol/g brain tissue, *P* > 0.05 vs. saline group) (Fig. 5b). Blood ammonia levels and brain ammonia levels in the LPS controls and D-GalN controls did not differ from those in the saline controls at all time points.

In mice with TNF-α/D-GalN-induced ALF, blood ammonia levels (Fig. 5c) and brain ammonia levels (Fig. 5d) began to increase at 2 h compared with those in the saline group (110.41 ± 21.59 μmol/L vs. 41.12 ± 16.90 μmol/L, *P* < 0.01) (2062.55 ± 619.516 vs. 1059.84 ± 152.48 nmol/g brain tissue, *P* < 0.01) and reached a peak level at 9 h (548.42 ± 178.16 μmol/L and 3710.93 ± 410.43 nmol/g brain tissue, respectively). Blood and brain ammonia levels at 12 h were decreased (392.58 ± 103.20 μmol/L and 2628.36 ± 791.85 nmol/g brain tissue) compared with those at 9 h, although they were significantly higher than those in the saline controls and TNF-α controls. At 24 h, blood ammonia levels remained higher than those in the saline controls (138.50 ± 23.34 μmol/L vs. 41.12 ± 16.90 μmol/L, *P* < 0.01), whereas brain ammonia levels decreased to normal level (1426.37 ± 500.44 nmol/g brain tissue).

After treatment with anti-TNF-α IgG antibodies, blood ammonia levels (Fig. 5e) at 12 h were significantly decreased in the LPS/D-GalN group (223.60 ± 91.40 μmol/L vs. 445.09 ± 184.60 μmol/L, *P* < 0.01), although they were higher than those in the saline group. Brain ammonia levels (Fig. 5f) were significantly decreased compared with those in the LPS/D-GalN 12 h group (1077.63 ± 133.69 nmol/g vs. 3511.83 ± 914.52 nmol/g brain tissue, *P* < 0.01), although the difference was not statistically significant compared with the saline controls.

### Rhcg expression in brain tissues

**Fig. 6:**
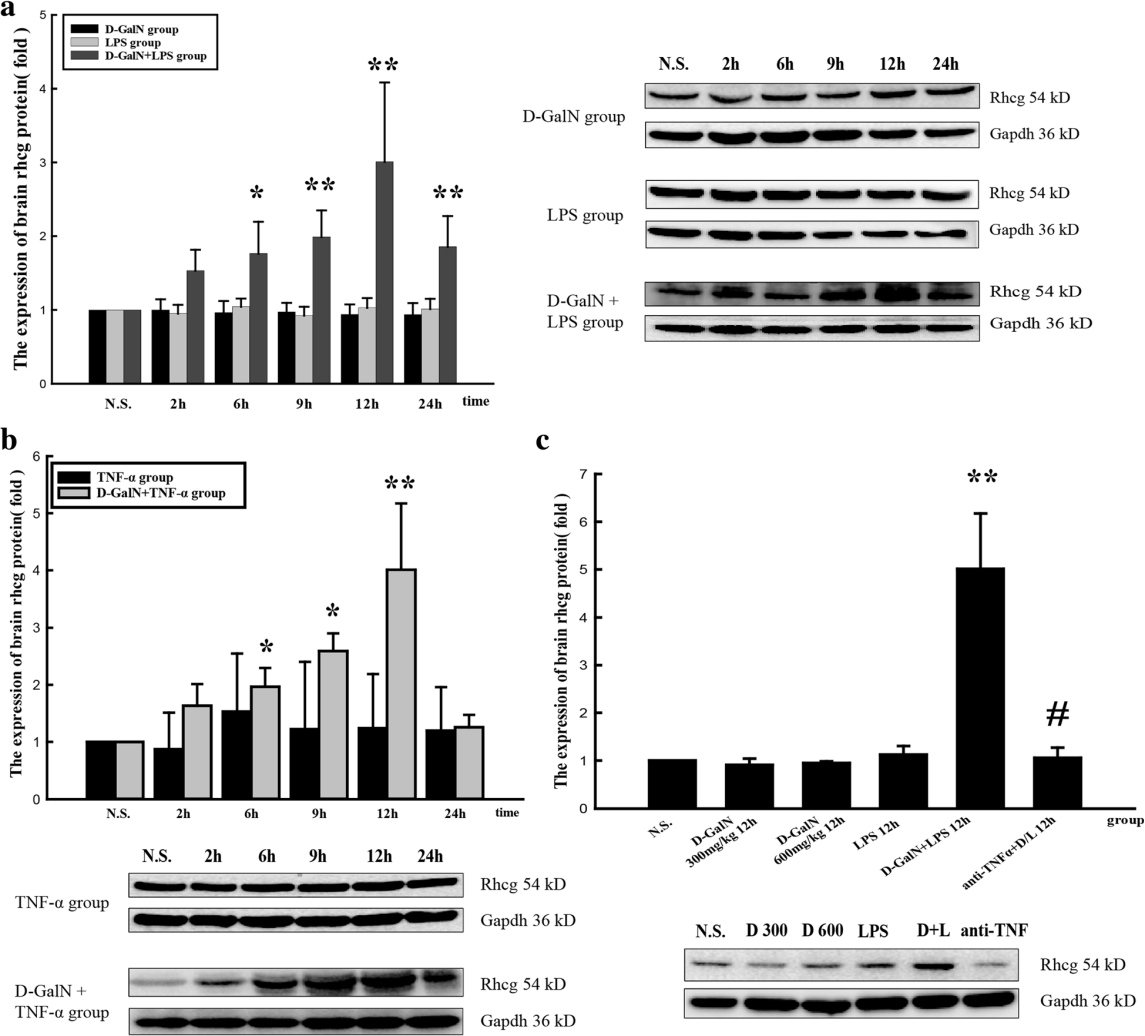
Expression of Rhcg protein in the brain. **a** Rhcg protein expression in LPS/D-GalN, D-GalN control, and LPS control groups. **b** Rhcg protein expression in TNF-α/D-GalN and TNF-α control groups. **c** Rhcg protein expression in the anti-TNF-α–IgG + LPS/D-GalN and other control groups at 12 h. Data represent the mean ± SEM (*n* = 5), *P < 0.05 vs. N.S. group, **P < 0.01 vs. N.S. group, #*P* < 0.01 vs. LPS/D-GalN 12 h group. N.S. normal saline, D-GalN d-galactosamine hydrochloride, LPS lipopolysaccharide, TNF-α tumor necrosis factor-alpha

Rhcg protein expression began to increase at 6 h and reached a peak at 12 h in both ALF groups injected with LPS/D-GalN (Fig. 6a) and TNF-α/D-GalN (Fig. 6b). Rhcg protein expression did not differ significantly between the brain tissues of D-GalN, LPS, and TNF-α control groups. Treatment with anti-TNF-α–IgG antibodies downregulated Rhcg expression (Fig. 6c).

**Fig. 7:**
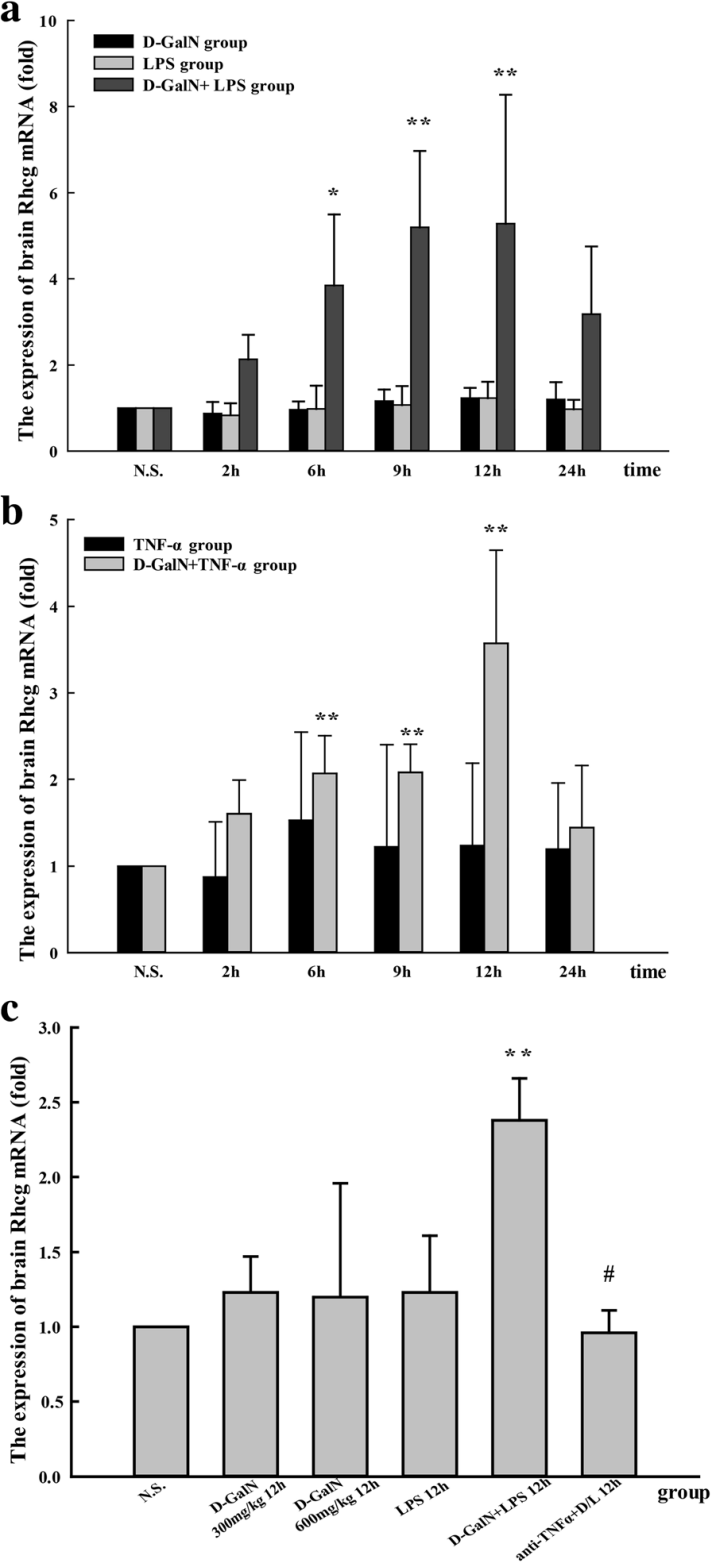
Rhcg mRNA expression in the brain. **a** Rhcg mRNA expression in LPS/D-GalN, D-GalN control, and LPS control groups. **b** Rhcg mRNA expression in TNF-α/D-GalN and TNF-α control groups. **c** Rhcg mRNA expression in anti-TNF-α–IgG + LPS/D-GalN and other control groups at 12 h. Data represent the mean ± SEM (*n* = 5), *P < 0.05 vs. N.S. group, **P < 0.01 vs. N.S. group, #*P* < 0.01 vs. LPS/D-GalN 12 h group. N.S. normal saline, D-GalN d-galactosamine hydrochloride, LPS lipopolysaccharide, TNF-α tumor necrosis factor-alpha

Rhcg mRNA expression began to increase at 6 h and reached a peak at 12 h in both ALF groups injected with LPS/D-GalN (Fig. 7a) and TNF-α/D-GalN (Fig. 7b). Rhcg mRNA expression did not differ significantly between the brain tissues of D-GalN controls, LPS controls, and TNF-α controls. Treatment with anti-TNF-α–IgG antibodies downregulated Rhcg expression (Fig. 7c).

Immunohistochemical analysis detected Rhcg expression in brain tissues of all groups, with especially strong expression in the LPS/D-GalN- and TNF-α/D-GalN-induced ALF groups. Treatment with anti-TNF-α–IgG antibody suppressed Rhcg expression in the LPS/D-GalN group (Fig. 4b, c). The immunohistochemical results were consistent with those obtained by the western blotting.

**Fig. 8:**
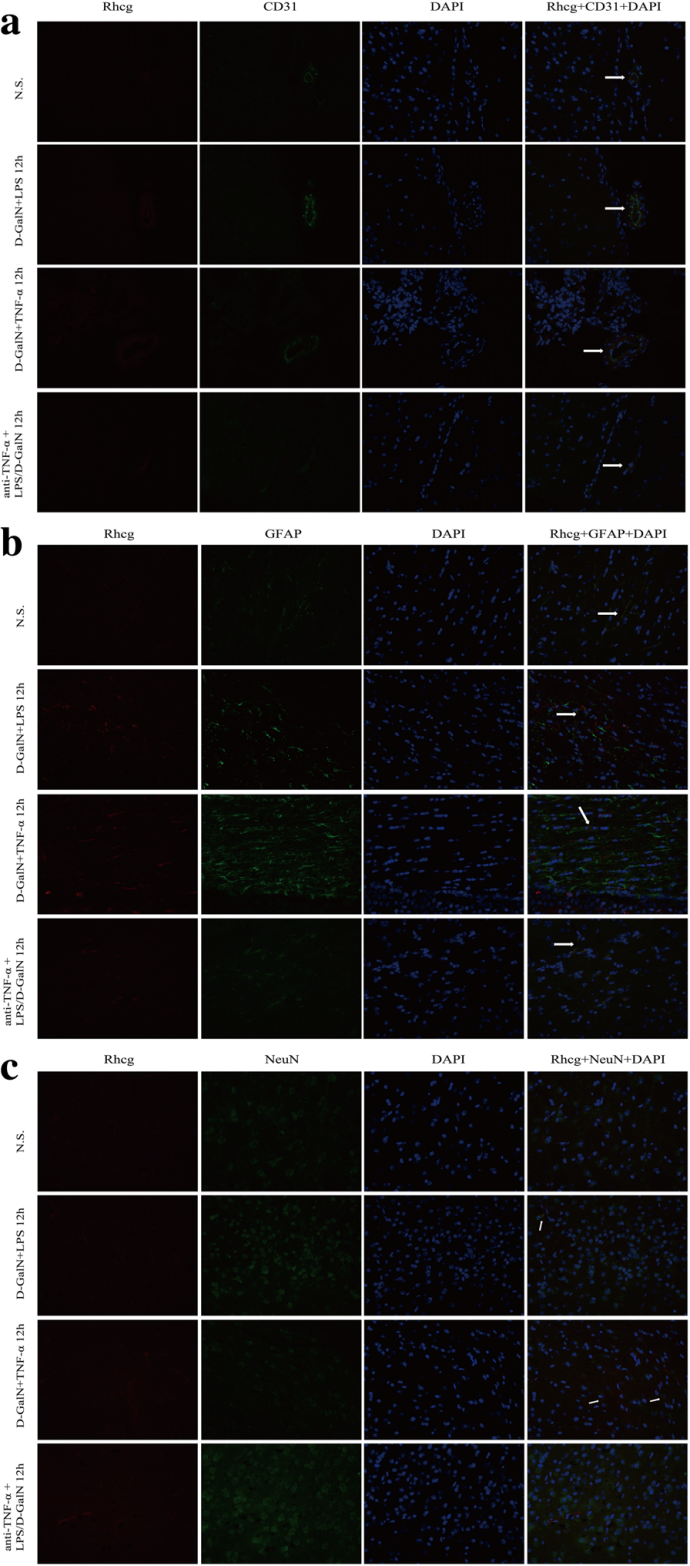
Representative images of immunofluorescence labeling of Rhcg and cell type-specific markers in brain tissues (× 400 original magnification). **a** Double-immunofluorescence analysis of Rhcg (red) and CD31 (green). **b** Double-immunofluorescence analysis of Rhcg (red) and GFAP (green). **c** Double-immunofluorescence analysis of Rhcg (red) and NeuN (green). Rhcg was detected in BMECs and astrocytes and at a lower level in neurons. N.S. normal saline, D-GalN, d-galactosamine hydrochloride, LPS lipopolysaccharide, TNF-α tumor necrosis factor-alpha, BMECs brain microvascular endothelial cells, GFAP glial fibrillary acidic protein, ALF acute liver failure, DAPI _4',6-diamidino-2-phenylindole_

Double-immunofluorescence staining of Rhcg and CD31 (Fig. 8a), GFAP (Fig. 8b), and NeuN (Fig. 8c) indicated that Rhcg is expressed in neurons and more robustly in BMECs and astrocytes.

## Discussion

The present study is the first to investigate the expression of the newly recognized ammonia transporter Rhcg in the murine brain. The murine brain expresses Rhcg at the mRNA and protein levels, and Rhcg expression is detected in brain microvascular endothelial cells, astrocytes, and neurons.

Since 2000, knowledge of the role of Rh glycoproteins in ammonia transport has increased considerably. Multiple experimental models examined their involvement in renal ammonia metabolism, and gene knock-out studies showed that they are critical for normal renal ammonia metabolism. However, the roles of these transporters in the brain remain unknown. A study on cancer-causing genes in glioblastoma identified Rhbg and Rhcg as candidates [30]. Hillmann et al. [31] showed that both RhBG and RhCG are expressed at the mRNA level in the NG108-15 cell line, which is a mouse neuroblastoma × rat glioma hybrid cell line that is frequently used as a model for neuronal cells. However, they did not detect RhBG and RhCG mRNA in 1321N1 astrocytoma cells. The authors hypothesized that the expression of NH_3_/NH_4_^+^ transporters may be one explanation for the fact that ammonium is not very toxic to neuronal cells, whereas it is highly toxic to astrocytes, which trap ammonia instead of extruding it.

The detection of Rhcg in the murine brain in our experiments differs from the results of Liu et al. [32]. They found that RhCG was abundantly expressed in the kidneys as well as in the brain, testis, placenta, pancreas, and prostate in human adult tissues. In mouse adult tissues, Rhcg was highly expressed in the kidney and testis but not in other tissues such as the brain [32]. The most likely explanation is that previous studies used Northern blot analysis, which is less sensitive for detecting mRNA expression than the real-time RT-PCR assay used in the current study. Similar observations regarding the ability to detect low levels of Rhbg and Rhcg mRNA by real-time RT-PCR, but not by Northern blot analysis, were reported previously in the lung [12] and intestinal tract, including the stomach, small intestine, and colon [11].

The pathophysiology of HE and brain edema in ALF are multi-factorial. As a potent neurotoxin, the release of ammonia from the failing liver reflects its critical role. To enter the brain, ammonia has to pass the blood-brain barrier. Decades ago, this barrier was thought to function as a semi-permeable membrane; however, this view is currently considered simplistic [5, 6, 33]. The luminal layer is formed by the endothelium, which is a highly active tissue; the brain endothelium has a higher concentration of mitochondria than any other endothelium in the body. The energy produced is used by a large number of membrane proteins that mediate the transport of ions and organic molecules. Since both the gas (NH_3_) and ion (NH_4_^+^) forms are capable of entering the cell, ammonia is considered a complex molecule in comparison with other weak acids and bases. Ammonia as a gas (NH_3_) is lipid soluble and therefore enters the brain through diffusion. In the present study, blood ammonia levels began to increase at 6 h, whereas brain ammonia levels began to increase at 2 h in the LPS/D-GalN group. In both the LPS/D-GalN and TNF-α/D-GalN groups, blood ammonia levels at 24 h were still higher than those in the saline controls, whereas brain ammonia levels had decreased to normal. These results suggest that ammonia enters the brain not only through diffusion. The results of the current study demonstrate that Rhcg is expressed in brain microvascular endothelial cells, astrocytes, and neurons, and that brain Rhcg expression is positively associated with blood and brain ammonia levels in ALF mice. The results indicated that Rhcg is involved in brain ammonia transport in brain capillaries in ALF. The specific molecular form of ammonia, NH_3_ versus NH_4_^+^, transported by Rh glycoproteins remains to be confirmed. Rhbg appears to transport both NH_3_ and NH_4_^+^, whereas its homolog Rhcg only appears to transport NH_3_ [34]. Bakouh et al. showed that RhCG-mediated NH_3_ and NH_4_^+^ fluxes vary during NH_4_Cl exposure, favoring the hypothesis of uncoupled fluxes or a bi-directional NH_3_ flux and unidirectional NH_4_^+^ flux (influx) [35, 36].

TNF-α is an important pleiotropic proinflammatory cytokine that is involved in many biological and pathological processes. Abundant evidence supports the critical role of TNF-α in the pathogenesis of ALF, especially in LPS/D-GalN-induced ALF [37–39]. In the present study, serum TNF-α levels reached a maximum at 2 h and were gradually restored at 6 h and 9 h, decreasing to normal values at 12 h and 24 h after LPS/D-GalN treatment. In the LPS control group, serum TNF-α levels also reached a maximum at 2 h and decreased dramatically to normal values at 6 h. LPS might induce macrophages and monocytes to release TNF-α. Our results indicated that the enhanced production of TNF-α induced by LPS/D-GalN was responsible for the development of ALF. When TNF-α was blocked with an antibody against TNF-α IgG, both the mortality and liver histopathology were significantly improved. Mice treated with TNF-α instead of LPS in conjunction with D-GalN showed similar histopathological characteristics to those of the LPS/D-GalN group. TNF gene polymorphisms significantly affect the outcome of ALF in humans and animals [40, 41]. TNF-α may contribute to this process through its receptors TNFR1 [42] and TNFR2 [43].

Several lines of evidence indicate that TNF-α could be involved in the pathogenesis of HE in ALF [24–27]. Patients with HE who are in ALF have elevated circulating levels of TNF-α [44]. A strong correlation between the circulating levels of TNF-α and those of ammonia was identified in these patients, which suggests a significant relationship between TNF-α and ammonia in patients with liver failure and HE. Research shows that TNF-α increases the diffusion of ammonia in central nervous system-derived endothelial cells [28], increasing the concentration of ammonia in the brain and consequently increasing ammonia toxicity to the central nervous system. TNF-α contributes to cell swelling in ammonia-sensitized cultured astrocytes [29]. We found that the elevation of serum TNF-α paralleled the increasing concentration of ammonia in the blood and brain tissues in D-GalN/LPS-induced ALF and was correlated with Rhcg expression in brain tissues. When TNF-α was blocked with an antibody against TNF-α IgG, Rhcg expression in ALF brain tissues was significantly downregulated; blood ammonia levels remained higher than in the saline group, but brain ammonia levels decreased to the baseline level. TNF-α is detected between 2 and 6 h from the start of the D-GalN+LPS treatment; blood and brain ammonia reaches a maximum level between 6 and 12 h; and Rhcg expression peaks between 9 and 12 h. The fact that TNF-α expression precedes that of the ammonia transporter Rhcg suggests that it may play a role in transcriptional or posttranslational upregulation of the *Rhcg* gene. The fact that the timing of Rhcg expression coincides with the ammonia peak in the brain indicates that Rhcg contributes to the accumulation of ammonia in the brain. These findings provide a likely mechanism for the exacerbation of brain edema or HE in patients with ALF. TNF-α may be involved in the development of brain edema in ALF by affecting brain ammonia through the modulation of Rhcg expression in the brain.

## Conclusions

In conclusion, the ammonia transporter Rhcg was expressed in brain tissues of C57BL/6 mice. Rhcg was expressed in brain microvascular endothelial cells, astrocytes, and neurons. Brain Rhcg expression was increased in LPS/D-GalN- and TNF-α/D-GalN-induced ALF mice and correlated with the blood and brain ammonia concentration. Rhcg may play an important role in ammonia transport in the central nervous system. TNF-α played an important role in LPS/D-GalN-induced ALF and the subsequent HE. Pretreatment with anti-TNF-α IgG antibody prevented the occurrence of ALF and reduced blood and brain ammonia levels in the LPS/D-GalN group. TNF-α might be involved in cytotoxic edema in ALF by regulating Rhcg expression in brain tissues, although the underlying mechanisms need to be further investigated.

## Abbreviations

ALF: Acute liver failure
ALT: Alanine transaminase
BBB: Blood–brain barrier
CE: Cerebral edema
D-GalN: d-Galactosamine hydrochloride
GFAP: Glial fibrillary acidic protein
HE: Hepatic encephalopathy
LPS: Lipopolysaccharide
RhBG/Rhbg: Rhesus glycoprotein B
RhCG/Rhcg: Rhesus glycoprotein C
TNF-α: Tumor necrosis factor-alpha
